# Room temperature ferromagnetism in metallic Ti_1−*x*_V_*x*_O_2_ thin films

**DOI:** 10.1039/c8ra06343e

**Published:** 2018-09-06

**Authors:** Ze-Ting Zeng, Feng-Xian Jiang, Li-Fei Ji, Hai-Yun Zheng, Guo-Wei Zhou, Xiao-Hong Xu

**Affiliations:** School of Chemistry and Materials Science of Shanxi Normal University, Key Laboratory of Magnetic Molecules and Magnetic Information Materials of Ministry of Education Linfen 041004 China jiangfx@sxnu.edu.cn; Research Institute of Materials Science of Shanxi Normal University, Collaborative Innovation Center for Shanxi Advanced Permanent Magnetic Materials and Techonology Linfen 041004 China

## Abstract

Transition metal doped TiO_2_ diluted magnetic semiconductors have attracted considerable interest due to their room temperature ferromagnetism. However, most TiO_2_ films are highly insulating, and thus the magnetic properties can not be controlled by tuning the carrier concentration. This will limit their application in controlling magnetization *via* electrical gating. Here, we deposit rutile Ti_1−*x*_V_*x*_O_2_ (*x* = 0.03 and 0.05) films with the thickness between 30 and 245 nm by the pulsed laser deposition technique, and observe an obvious room temperature ferromagnetic behavior in all films. The high resolution X-ray photoelectron spectroscopy results indicate that V substituting Ti^4+^ ions in the TiO_2_ lattice, with the +3 valence state having two unpaired d electrons, is responsible for the local spin. More importantly, the systemic investigations of transport properties for Ti_1−*x*_V_*x*_O_2_ films reveal that the films are n-type and have metallic conductivity with a carrier density of about 10^20^/cm^3^. Further studies suggest that the oxygen vacancies play a dual role of contributing to the metallic conductivity of the Ti_1−*x*_V_*x*_O_2_ films, and also providing the free electrons to mediate the long-range ferromagnetic coupling between two magnetic polarons. These findings may offer promise for gate-tunable ferromagnetism in future semiconductor spintronics.

## Abstract 2

Transition metal doped TiO_2_ diluted magnetic semiconductors have attracted considerable interest due to their room temperature ferromagnetism.

## Graphical abstract

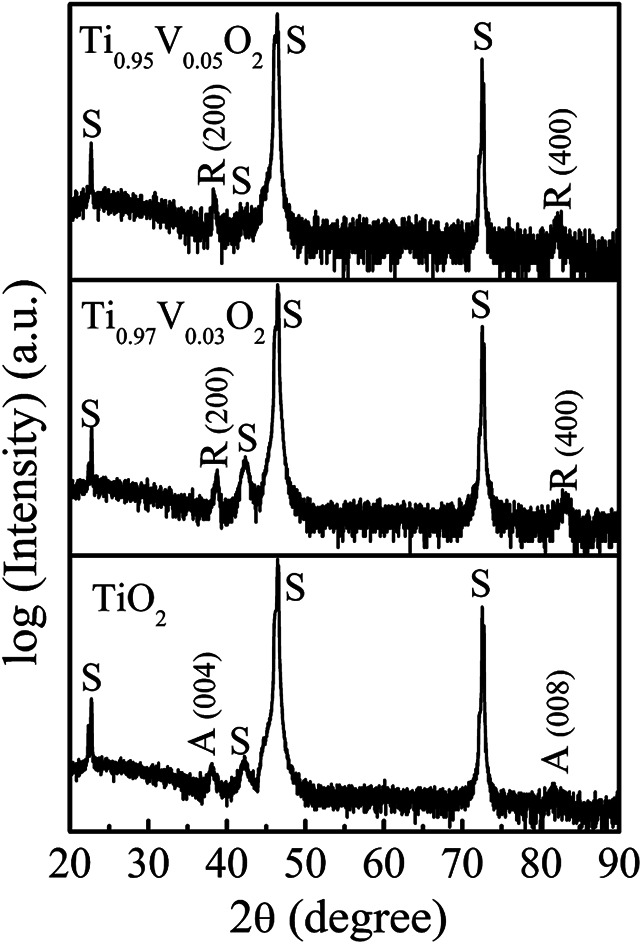

## Introduction

Diluted magnetic semiconductors (DMSs) have attracted enormous attention due to their potential applications in spintronic devices.^1–3^ To date, the III–V based DMSs, such as Mn doped GaAs, have been well studied, however, the low values of the Curie temperature (*T*_C_) hindered their application at room temperature.^4,5^ An important step forward in the field was the theoretical prediction by Dietl *et al.* of high temperature ferromagnetism in Mn doped ZnO.^6^ Subsequently, the Co doped TiO_2_ thin films with the anatase structure were reported to be ferromagnetic even above 400 K with a magnetic moment of 0.32 *μ*_B_ per Co atom*.*^7^ Since then, transition metal (TM) doped TiO_2_ DMS has attracted particular interest as TiO_2_ has many advantages, such as low cost, good dielectric properties and high chemical stability.^8–12^ However, due to the low solubility of TMs in TiO_2_, extrinsic effects, such as magnetic clusters and impurity phases, are often responsible for the observed ferromagnetism.^13–15^ Furthermore, many studies have focused on the effects of the methods and growth conditions on the structural and magnetic properties of TiO_2_ DMS,^16–20^ and it is also found that most TiO_2_ films doped with different transition elements are highly insulating.^21–26^ For example, Griffin *et al.*^21^ grew a series of anatase Co:TiO_2_ films by RF magnetron sputtering, and obtained a saturation magnetic moment of 1.1 *μ*_B_/Co, while all films were highly insulating. Sharma *et al.*^25^ showed that the Mn-doped TiO_2_ films prepared by the spray pyrolysis technique also exhibited the highly insulating nature with the resistivity of almost 10^7^ Ω cm. It is noted that although some reports demonstrate that the incorporation of nonmagnetic element Nb and Ta in TiO_2_ can lead to metallic electrical conduction,^27–32^ the origin of magnetic moments is attributed to cationic vacancies.^31,32^ A DMS, containing a dilute concentration of magnetic ions imbedded in the host lattices, is characterized by the free carriers mediated exchange interactions between the magnetic ions. In such systems, the magnetization can be controlled by tuning the carrier density *via* electrical gating. In order to meet this criterion, it is essential to obtain the conductive TiO_2_ films.

In this work, we obtain the metallically conductive Ti_1−*x*_V_*x*_O_2_ films with different thickness by using the pulsed laser deposition (PLD) technique with precise control of oxygen pressure at 3 × 10^−3^ mTorr. The structural, composition and magnetic results suggest that the observed room temperature ferromagnetism in Ti_1−*x*_V_*x*_O_2_ films is intrinsic. Further studies indicate that the oxygen vacancy not only contributes to the metallic conductivity of the Ti_1−*x*_V_*x*_O_2_ films, but also it provides the free electrons to mediate the long-range ferromagnetic coupling between two magnetic polarons.

## Experimental method

The Ti_1−*x*_V_*x*_O_2_ films (*x* = 0.03 and 0.05) with the thickness of 30–245 nm were grown on SrTiO_3_ (100) substrate by the PLD technique at a temperature of 800 °C and an oxygen partial pressure of 3 × 10^−3^ mTorr. The laser pulses were supplied by a KrF excimer source (*λ* = 248 nm) with an energy density of 2.5 J per cm^2^ per shot and a frequency of 10 Hz. The nominal Ti_1−*x*_V_*x*_O_2_ targets were prepared by a solid-state reaction method using TiO_2_ (99.99%) and V_6_O_13_ (99.97%) powders, and they were ablated for 5 minutes to eliminate surface contamination before deposition. After deposition, the films were annealed *in situ* for 30 minutes, and then cooled down to room temperature slowly at the same oxygen pressure. The crystal structures of the films were analyzed by *θ*–2*θ* X-ray diffraction (XRD) with using Cu Kα radiation (*λ* = 0.15406 nm). The chemical composition was determined by X-ray photoelectron spectroscopy (XPS) with a monochromatic Al Kα radiation as the X-ray source. The magnetic properties were measured by a superconducting quantum interference device (SQUID) magnetometer. The transport properties of the films were determined in the four-point probe configuration using a Quantum Design physical properties measurement system (PPMS) as a function of temperature.

## Results and discussion

**Fig. 1:**
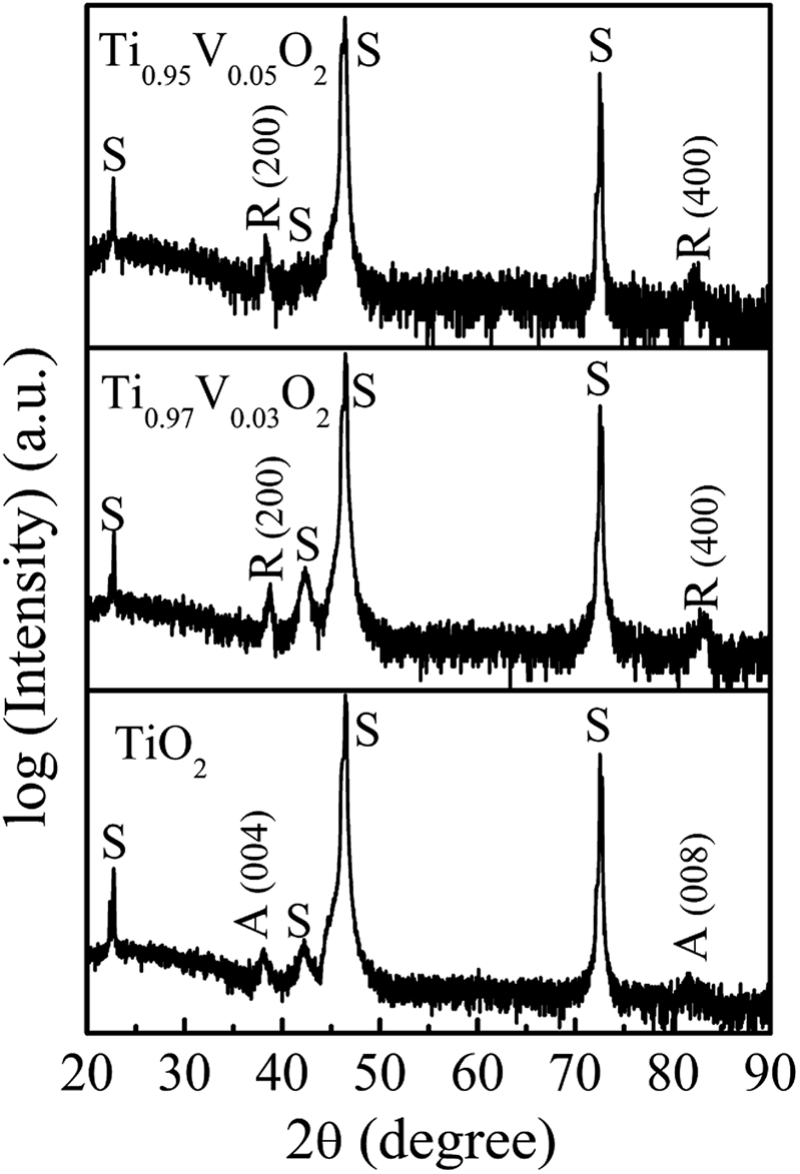
The XRD patterns of Ti_1−*x*_V_*x*_O_2_ films. The peaks marked with “S”, “A” and “R” correspond to the peaks of SrTiO_3_ substrates, anatase TiO_2_ and rutile TiO_2_, respectively.

Fig. 1 shows the XRD patterns of Ti_1−*x*_V_*x*_O_2_ films (*x* = 0.03 and 0.05) with the thickness of about 100 nm. Here, the XRD pattern for pure TiO_2_ film deposited as the same condition is also placed at the bottom of figure for comparison. The spectra are plotted on a logarithmic scale to discern any minor secondary phase with small intense reflections. The results show that the Ti_1−*x*_V_*x*_O_2_ films are epitaxial with single-phase rutile phase character, with only (200) and (400) reflections detectable. It is noted that the undoped TiO_2_ film is epitaxial and of anatase phase with the (00*l*) orientation. It has been proposed that two Ti–O bonds break in the anatase structure, allowing the rearrangement of the Ti–O octahedra, which leads to a smaller volume and the rutile phase.^33^ The breaking of these bonds is accelerated by the lattice disruptions, which can be introduced by the presence of dopant ions, the oxygen vacancies, and the method of synthesis.^34^ In our study, the Ti_1−*x*_V_*x*_O_2_ films were deposited at a high vacuum (3 × 10^−3^ mTorr), resulting in a large amount of oxygen vacancies in the films. This will presumably reduce the strain energy that must be overcome before the rearrangement of the Ti–O octahedral can occur,^33^ and thus promotes the phases transformation.

**Fig. 2:**
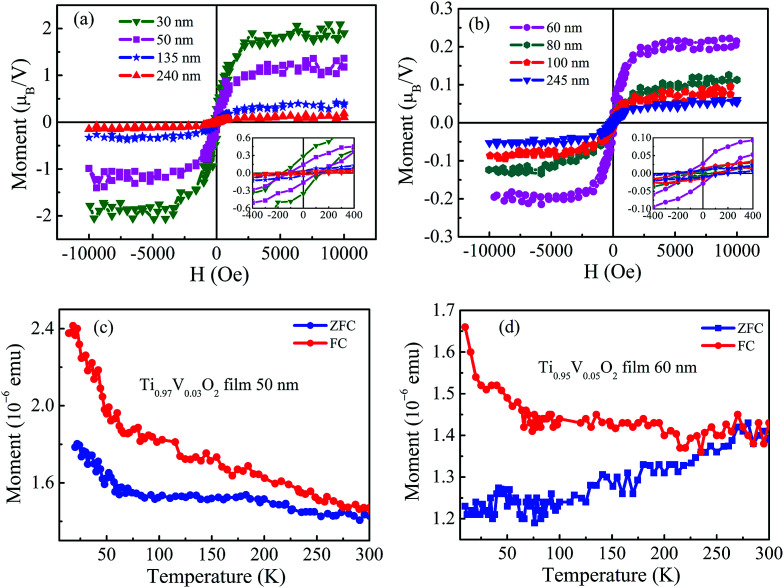
Room temperature M − H loops of Ti_0.97_V_0.03_O_2_ (a) and Ti_0.95_V_0.05_O_2_ (b) films, respectively, with different thickness. The insets show the magnified M − H loops of the films. (c) and (d) The ZFC/FC curves of the Ti_0.97_V_0.03_O_2_ and Ti_0.95_V_0.05_O_2_ films with thickness of 50 and 60 nm, respectively.

The Ti_1−*x*_V_*x*_O_2_ films with different thickness exhibit the obvious room temperature ferromagnetism. The in-plane room temperature magnetic hysteresis (M − H) loops of the films are shown in Fig. 2(a) and (b). The diamagnetic contribution from the substrate is subtracted from the loops. The insets show the magnified M − H loops of the films. The saturation magnetizations (*M*_s_) of the Ti_0.97_V_0.03_O_2_ films with thickness of 30, 50, 135 and 240 nm are 1.9, 1.2, 0.4 and 0.1 *μ*_B_/V, and their coercive field (*H*_c_) are 165, 110, 75, and 30 Oe, respectively. For the Ti_0.95_V_0.05_O_2_ films, the maximum values of *M*_s_ and *H*_c_ obtained in the thinner films are 0.5 *μ*_B_/V and 120 Oe, respectively. It clearly shows the reduction in *M*_s_ and *H*_c_ with increasing thickness. The observation of the larger *M*_s_ value in thinner film could be attributed to more structural or surface defects in the films.^35^ For comparison of Fig. 2(a) and (b), it can be found that the values of *M*_s_ for Ti_0.95_V_0.05_O_2_ films are smaller than those of Ti_0.97_V_0.03_O_2_ films at the same thickness. This could be assigned to an increase in the antiferromagnetic coupling between V ions at shorter separations.^36^Fig. 2(c) and (d) display the zero-field cooled (ZFC) and field-cooled (FC) magnetization curves at a field of 100 Oe for the Ti_1−*x*_V_*x*_O_2_ films. There is no evidence of the blocking temperature in the whole temperature range of 10–300 K, suggesting that the tiny ferromagnetic nano-clusters are not present in the films.^37,38^ Moreover, the ZFC/FC curves are distinctly separated from each other without any phase transition from 10 to 300 K, indicating that the *T*_C_ of the Ti_1−*x*_V_*x*_O_2_ films is higher than 300 K.

**Fig. 3:**
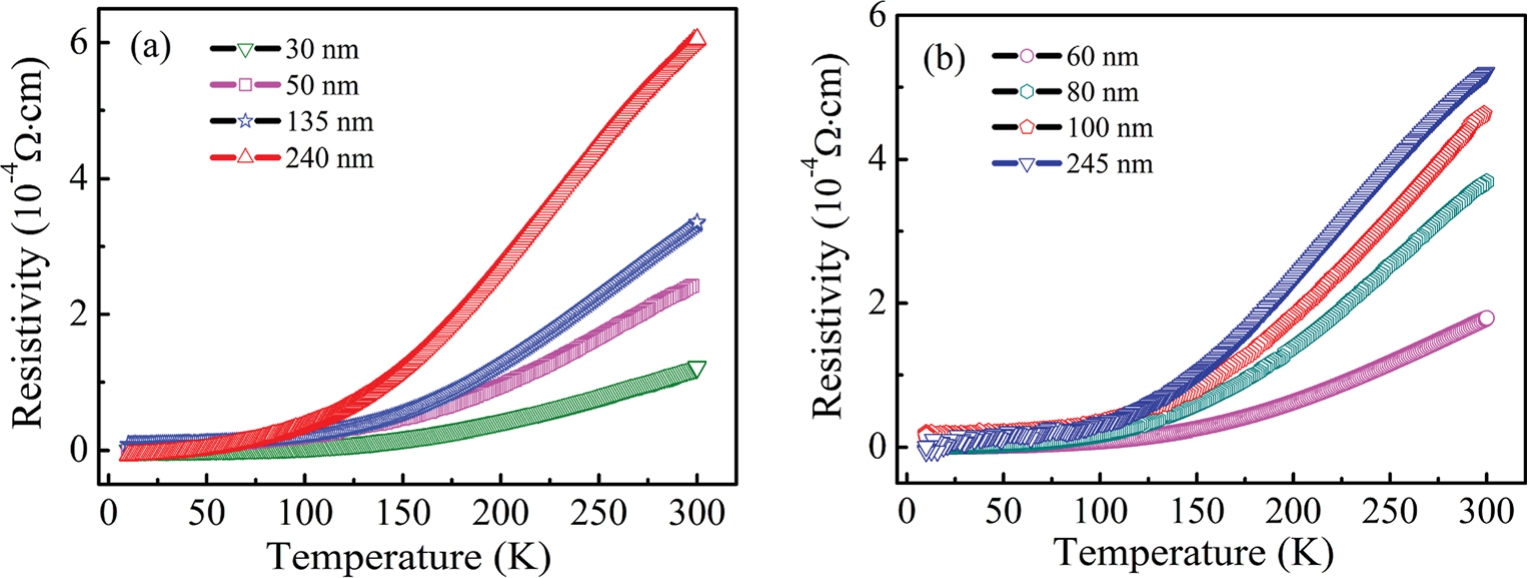
The temperature dependent resistivity as a function of thickness of Ti_0.97_V_0.03_O_2_ (a) and Ti_0.95_V_0.05_O_2_ (b) films.

The four-point probe geometry was used to obtain the transport properties of the Ti_1−*x*_V_*x*_O_2_ films. The results indicate that all of the films show n-type conductivity and the carrier concentration is about 10^20^/cm^3^. The temperature (*T*) dependence of resistivity (*ρ*) is measured down to 10 K, which is shown in Fig. 3. All the resistivity *versus* temperature curves show positive slope, indicating an metallic conductivity, and the resistivity slightly increases as the films thickness increases. Hong *et al.*^39^ deposited the V-doped TiO_2_ films on LaAlO_3_ substrates by the PLD method, and they found that the films were semiconductors and the resistivity at room temperature was as high as 10^7^ Ω cm, which is very different from our films. This may be due to the influence of preparation or processing conditions and the resulting defects on the transport properties of V–TiO_2_ films. Osorio-Guillén *et al.*^40^ studied theoretically the electronic behaviors in V doped anatase TiO_2_, and showed that V_Ti_ introduced deep levels in the gap due to the low 3d energy of the V atoms, resulting in the nonconductive for V-doped TiO_2_.

**Fig. 4:**
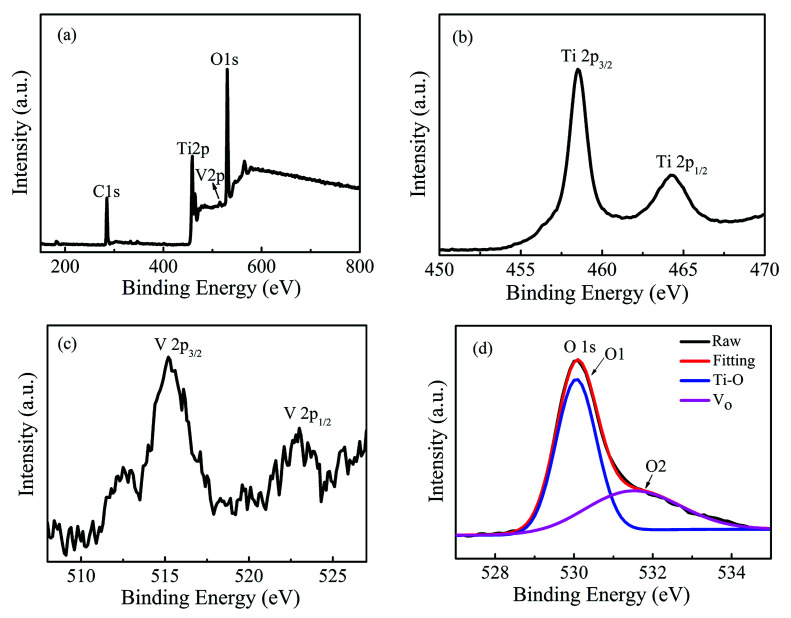
XPS spectra of Ti_0.95_V_0.05_O_2_ film with the thickness of 180 nm: the survey spectra (a) and high-resolved spectra of Ti 2p (b), V 2p (c) and O 1s (d), respectively.

Now, the possible origins of room temperature ferromagnetism and metallic behavior in the Ti_1−*x*_V_*x*_O_2_ films will be explored. If some portion of V is in the +3 or +4 valence states, or Ti is in the +3 valence state, and then the V^3+^, V^4+^ or Ti^3+^ will act as a localized spin, which is prerequisite to induce the magnetic ordering.^41^ The n-type donors of V^5+^ and oxygen vacancies may contribute to the metallic conductivity of the films. In order to examine these possibilities, the XPS measurement was performed to determine the chemical states of Ti, O and V in the Ti_1−*x*_V_*x*_O_2_ films. Fig. 4(a) shows the XPS survey spectra of Ti_0.95_V_0.05_O_2_ film with the thickness of 180 nm. No additional peaks corresponding to secondary phases are detected, which is in accordance with the XRD and ZFC/FC measurements. Fig. 4(b) shows the Ti 2p spectrum for the same sample with Ti 2p_3/2_ and Ti 2p_1/2_ located at 458.5 and 464.3 eV, respectively, suggesting that Ti is in the +4 state.^25,42^ The peak separation between the 2p_3/2_ and 2p_1/2_ lines is 5.8 eV, which is also consistent with the Ti^4+^ oxidation state.^43,44^ The binding energies of V 2p_3/2_ and V 2p_1/2_ shown in Fig. 4(c) are 515.2 and 523.4 eV, respectively, indicating that V is in the +3 state.^45,46^Fig. 4(d) displays the spectra of O1s, which are divided into two peaks, referred to as O1 and O2. The peaks near 530.2 and 531.9 eV correspond to the binding energy of lattice oxygen in TiO_2_ and oxygen defects, respectively.^47^ It can be seen that an amount of oxygen vacancies exists in the Ti_0.95_V_0.05_O_2_ film, which can be ascribed to the films deposited at a very low deposition oxygen pressure (3 × 10^−3^ mTorr). Additionally, the substitution of Ti^4+^ by V^3+^ ions will also increase the concentration of oxygen vacancies due to the necessity for the charge balance. Based on the XPS results, it is reasonable to claim that in the Ti_1−*x*_V_*x*_O_2_ films the V^3+^ ions provide the local magnetic moment and the metallic conductivity can be attributed to the ionized donors of oxygen vacancies. This result is consistent with the theoretical calculations by Osorio-Guillén *et al.* that V dopants could convert nonmagnetic TiO_2_ into a ferromagnet as V_Ti_ can introduce a partially occupied, spin-polarized level, which could promote ferromagnetism.^40^

There are different mechanisms for ferromagnetic coupling in the literature for TiO_2_-based DMS, such as carrier-mediated exchange^48^ and bound magnetic polaron (BMP) model.^49^ Tian *et al.*^36^ speculated that the ferromagnetic coupling between V ions mediated by oxygen vacancies at interfaces may account for the observed room temperature ferromagnetism in V-doped TiO_2_ nanoparticles. Hong *et al.*^50^ reported the large value of magnetic moment of 4.2 *μ*_B_/V for the V:TiO_2_ films and suggested that the room temperature ferromagnetism did not come from V clusters but from V-doped TiO_2_ matrix. Du *et al.*^51^ used a first principles to study the magnetic properties of anatase Ti_1−*x*_V_*x*_O_2_, and showed that the oxygen vacancy induced magnetic polaron could produce long-range ferromagnetic interaction between largely separated V impurities. In the present work, V is chosen as a dopant because it is impossible to form any ferromagnetic secondary phase of V metal and V oxide, ruling out the extrinsic origin of the ferromagnetism. Indeed, the XRD, XPS and ZFC/FC results suggest that the observed room temperature ferromagnetism in Ti_1−*x*_V_*x*_O_2_ films is intrinsic. Moreover, there are amount of oxygen vacancies in the Ti_1−*x*_V_*x*_O_2_ films, and the films exhibit the metallic behavior (Fig. 3) with the high carrier concentration of 10^20^/cm^3^. In this regard, we propose that the doped V^3+^ ions ferromagnetically couple with the electrons trapped by the oxygen vacancies, and form the BMPs, the carriers mediated the long-range ferromagnetic coupling between the magnetic polarons is a more possible mechanism in Ti_1−*x*_V_*x*_O_2_ films. This is in agreement with our previous theoretical results.^52^

## Conclusions

In summary, we have prepared rutile Ti_1−*x*_V_*x*_O_2_ (*x* = 0.03 and 0.05) films with different thickness by using the pulsed laser deposition technique, and observed ferromagnetism at room temperature. The structural, composition and magnetic results suggested that the room temperature ferromagnetism in Ti_1−*x*_V_*x*_O_2_ films was intrinsic. More importantly, the Ti_1−*x*_V_*x*_O_2_ films showed n-type and metallic conductivity. Further studies indicate that the oxygen vacancy not only contributes to the metallic conductivity of the Ti_1−*x*_V_*x*_O_2_ films, but also it provides the free electrons to mediate the long-range ferromagnetic coupling between two magnetic polarons.

## Conflicts of interest

There are no conflicts to declare.

## Supplementary Material
